# 

**Published:** 1892-12

**Authors:** 


### CONTENTS.

### COMMUNICATIONS.

Antizymotics, or Agents which Prevent Fermentation and Putrefaction .......... 569
Wash for Chapped Hands .......... 579
The Importance of a Knowledge of Bacteriology to the Dentist .......... 580
Dentistry in the Forties .......... 583
Combining Business Methods with Professional Methods in Dealing with our Patients .......... 586
Difficult Dental Prosthesis .......... 591
The Treatment of Physical Pain .......... 596

### PROCEEDINGS.

Michigan Dental Association .......... 597

### SELECTIONS.

Uses for the Oxysulphate of Zinc .......... 606
Position in Sleep .......... 609

### EDITORIAL.

A Correction .......... 611
Bibliographical .......... 611
For the Worlds Fair .......... 612

